# Editorial: Microbial Ecology in Reservoirs and Lakes

**DOI:** 10.3389/fmicb.2020.01348

**Published:** 2020-06-26

**Authors:** Haihan Zhang, Raju Sekar, Petra M. Visser

**Affiliations:** ^1^Shaanxi Key Laboratory of Environmental Engineering, Key Laboratory of Northwest Water Resource, Environment and Ecology, Ministry of Education, Xi'an University of Architecture and Technology, Xi'an, China; ^2^School of Environmental and Municipal Engineering, Xi'an University of Architecture and Technology, Xi'an, China; ^3^Department of Biological Sciences, Xi'an Jiaotong-Liverpool University, Suzhou, China; ^4^Department of Freshwater and Marine Ecology, Institute for Biodiversity and Ecosystem Dynamics, University of Amsterdam, Amsterdam, Netherlands

**Keywords:** reservoirs, lakes, surface water, sediments, DNA sequencing, CARD-FISH, ARGs, freshwater

In freshwater ecosystems, microbes play major roles in global energy fluxes and diverse biogeochemical (C, N, P, S, and other elements) cycling pathways of deep reservoirs and shallow lakes (Liu et al., 2015, 2019; Savvichev et al., 2018). Compared to oligotrophic, stable deep ocean ecosystems, these freshwater bodies host distinct microbial communities that are associated with water and sediments (Hutchins and Fu, 2017). The aquatic microbial populations in freshwater and oceans may be largely driven by changes in the nutritional status of the water bodies due to variations in the hydrological regime and climate change (He et al., 2015; Beall et al., 2016; Hayden and Beman, 2016). Recently, thanks to the fast development of high-throughput sequencing technology (Shade et al., 2012) and bioinformatics combined with functional genomics (Berg et al., 2016), an unexpected diversity of functional microbes was unveiled in reservoirs and lakes (Preheim et al., 2016; Xue et al., 2018; Yan et al., 2020).

The Frontiers Research Topic—*Microbial Ecology in Reservoirs and Lakes*—invited contributions in the following areas: (a) Relationship between water quality parameters and microbial community composition; (b) Functional microbial communities in water and sediments; (c) Effects of hydrological regimes (e.g., thermal stratification, rainstorm, water level) on dynamics of microbial communities; and (d) Algal blooms and their interactions with other microbial communities. Finally, special emphasis was placed on modeling of ecosystem-based water quality data and microbial community composition using DNA sequencing techniques.

A total of 21 articles have been published in this Research Topic and combined in this e-book to highlight the new findings on diverse aspects and recent advances in microbial ecology (e.g., community diversity and distribution of prokaryotes and eukaryotes in various freshwater environments, quantification of microbes that are associated with blooms, antibiotic resistance, and fecal contaminations). The research reported in these articles was carried out in eutrophic, mesotrophic, and oligotrophic reservoirs and lakes located in different regions of China, Canada, Japan, Europe, and the USA. The aim of this Editorial article is to summarize the new findings reported in these articles, to broaden our understanding of the composition, diversity, abundance, dynamics, and function of microbes in freshwater ecosystems.

In this topic, Shen et al. used metagenomics workflow to reveal the relationships between the trophic status and planktonic microbiota in freshwater lakes on Yun-Gui Plateau, China. The microbial communities in the eutrophic and mesotrophic-oligotrophic lake ecosystems showed a large difference in community structure. The authors addressed that the overall differences in the genetic potential for elemental cycling and metabolic functions were closely correlated to the divergence of the microbial community. Wu Y.-F. et al. employed high-throughput sequencing to investigate microbial succession during anaerobic decomposition of *Microcystis* on eutrophic sediments collected from Mei Liang Bay of Lake Taihu. The results showed that addition of *Microcystis* to the sediment induced phylogenetic clustering and structure instability of the sediment microbial community. Cruaud et al. showed that eukaryotic microbial communities had clear seasonal patterns in a Canadian river which were likely caused by the changes of environmental conditions. At the same time, a probable contribution of the bacterial community to the temporal distributions of the protist community structure was supported by potential interplays with the bacterial community composition.

The distribution of kinetoplastids in deep water layers during summer stratification was revealed using group-specific catalyzed reporter deposition and fluorescence *in situ* hybridization (CARD-FISH) probes and 18S rRNA gene sequencing by Mukherjee et al.. The results indicated that kinetoplastids were widely distributed in deep waters. Unexpectedly, the authors found the presence of diplonemids, a sister group of kinetoplastids. Colombet et al. reported the discovery of “Aster-Like Nanoparticles (ALNs)” in pelagic environments. This study shows that ALNs are novel and abundant in aquatic ecosystems and that not all virus-like particles detected in aquatic systems are necessarily viruses. Moreover, the study concluded that there may be more unknown ecologically important ultra-small particles in the aquatic systems. Cai et al. studied variations of δ^15^NPN and δ^15^NTDN during thermal stratification and their relationships with the environmental factors and phytoplankton in Lianhe Reservoir, China. The results revealed that δ^15^ NPN and δ^15^ NTDN changed with seasonal thermal cycling; moreover, cell density was the main factor that regulated the nitrogen stable isotope distribution. Davenport et al. undertook a meta-transcriptomic approach to reveal the temporal changes in the metabolic functions of *Microcystis* spp. During the bloom periods, the results demonstrated that there was different gene expression patterns between samples collected during the day or night. Besides, the partition of *Microcystis* gene expression depended on both circadian regulation and changes in environmental physico-chemical factors.

To better understand the spatial and seasonal variations of picophytoplankton communities in aquatic ecosystems, Shi et al. performed high-throughput sequencing to study the abundance and diversity of photosynthetic picoeukaryotes (PPEs) and phycoerythrin-rich picocyanobacteria (PE-cells) in Lake Fuxian, China. *Synechococcus* was the major PE-cell type, with a relatively similar abundance throughout the year, except for a decrease in summer. The authors showed that seasonal changes significantly influenced the composition, diversity, and abundance of PPE -cells. Jiang et al. analyzed the microbiota and their relationships with temperature changes and other environmental variables within a decadal period in five alpine lakes using 16S rRNA gene deep-amplicon sequencing. The results revealed that temperature, nutrients, and dissolved organic carbon had a significant effect on the bacterial community composition. Bomberg et al. investigated the composition and the metabolic features of the microbial communities in water bodies separated by permafrost collected in the area of Kangarlussuaq, Western Greenland. This study showed that highly diverse microbial communities existed in different cold Greenlandic aqueous environments, and showed clear patterns in the microbial communities according to habitats, with unique metabolic characteristics.

Wu K. et al. investigated the potential drivers of the relative abundance of bacterial communities and their relationship with water-depth in Lake Lugu located in Southwest China. The results showed that water depth was the most important factor that affected the relative abundance of 11 dominant bacterial species. Meanwhile, other physical, chemical, and biological variables also had a greater impact on the abundance of some bacterial species.

Fang et al. used high-throughput approaches to investigate the dynamics of antibiotic resistance genes (ARGs) in relationship with the microbial communities and environmental patterns in a subtropical urban reservoir in China by high-frequency sampling. The results indicated that the bacterial community had a seasonal pattern, while the ARGs composition did not change seasonally; moreover, the bacterial abundance and the community diversity were much more strongly correlated with environmental factors than ARGs. Palacin-Lizarbe et al. quantified guilds of four genes related to the N-transformation pathway in benthic habitats of 11 mountain lakes in Spain to reveal the effects of nitrogen deposition on microbial-driven patterns in oligotrophic freshwater ecosystems. They pointed out that those microbes of different gene types live in different water-depth layers leading to two different responses to high atmospheric N deposition in oligotrophic lakes of different trophic status.

Nakatsu et al. studied the impact of riverine microbial impact on Lake Michigan. The study assessed if Grand Calumet River was a source of bacterial contamination at different sites in Lake Michigan, and whether the feces associated bacteria in the samples were related to the pathogen indicator species *Escherichia coli*. The results showed that there were significant differences in bacterial communities at different sites, and environmental factors had a significant effect on this difference. At the same time, there was a significant positive correlation between fecal-related bacteria and pathogen-indicator organisms. Vadde et al. evaluated the most suitable microbial source tracking qPCR assays for detecting host-associated fecal pollution across the Tiaoxi River in the Taihu watershed. Their experiments indicated that several locations in the Tiaoxi River are heavily polluted by fecal contamination and this correlated well with the land-use patterns. Moreover, Xu et al. collected historical data of harmful algal blooms (HABs) events that occurred in the Bei Bu Gulf in China, and investigated the pattern of HABs in order to predict the future trends. This paper confirmed that over the past several years, HABs became progressively worse. Liu et al. evaluated the effects of different DNA extraction kits on the abundance and distribution of rare and abundant plankton taxa in the surface layers of a reservoir. Their experiment showed that the use of different DNA extraction kits had greater impact on rare taxa than on abundant taxa. Besides, different DNA extraction kits had their own advantages when studying different microbes. Furthermore, Monchamp et al. used high-throughput sequencing approaches to study the diversity and composition of non-photosynthetic cyanobacteria (NCY) in sediment cores of 10 lakes of the European peri-Alpine region. The authors found that different lake had different NCY communities, while there was no significant change in the diversity of NCY combinations within and between lakes over the past 100 years. In seven European perialpine lakes, Okazaki et al. investigated the richness and composition of CL500-11(Phylum Chloroflexi) by means of CARD-FISH. This paper explored a wide habitat range of CL500-11 in ultra-oligotrophic lakes or mesotrophic lakes. In summary, we expect that these papers will stimulate new discussions and investigations on the microbial ecology of freshwater reservoirs and lakes. The results will improve our understanding of the molecular microbial ecological characteristics of freshwater ecosystems. From a more applied perspective, it also shows the basic database for environmental and water resource management to increase the health status of reservoirs and lakes by using some artificial strengthening technologies (e.g., water lifting aerators) to enhance the microbial community metabolic activity in drinking water reservoirs ecosystems, and supply cleaner and healthier drinking water for consumers.

## Author Contributions

All authors listed have made a substantial, direct and intellectual contribution to the work, and approved it for publication.

## Conflict of Interest

The authors declare that the research was conducted in the absence of any commercial or financial relationships that could be construed as a potential conflict of interest.
